# Heterogeneity and therapeutic implications of cancer-associated fibroblasts in lung cancer: Recent advances and future perspectives

**DOI:** 10.1016/j.pccm.2024.08.009

**Published:** 2024-10-24

**Authors:** Chunhui Yang, Wenwen Liu, Charles A. Powell, Qi Wang

**Affiliations:** a Department of Clinical Laboratory, The Second Hospital, Dalian Medical University, Dalian, Liaoning 116023, China; b Translational Research Center for Lung Cancer, The Second Hospital, Dalian Medical University, Dalian, Liaoning 116023, China; c Division of Pulmonary, Critical Care, and Sleep Medicine, Icahn School of Medicine at Mount Sinai, New York, NY 10029, USA; d Department of Respiratory Medicine, The Second Hospital, Dalian Medical University, Dalian, Liaoning 116023, China

**Keywords:** Lung cancer, Cancer-associated fibroblasts, Tumor microenvironment, Therapeutic resistance, Biomarker

## Abstract

Lung cancer is a leading cause of cancer-related mortality. The tumor microenvironment is a complex and heterogeneous cellular environment surrounding tumor cells, including cancer-associated fibroblasts (CAFs), blood vessels, immune cells, the extracellular matrix, and various cytokines secreted by cells. CAFs are highly heterogeneous and play crucial roles in lung cancer. This review highlights recent advances in the understanding of CAFs in lung cancer, focusing on their heterogeneity and functions in tumorigenesis, progression, angiogenesis, invasion, metastasis, therapy resistance, tumor immune suppression, and targeted therapy responses. Additionally, we explore the underlying mechanisms and the potential of CAFs as a target in the development of innovative therapies for lung cancer.

## Introduction

Lung cancer is the leading cause of cancer-related deaths worldwide, accounting for 11.6% of all cancer cases and 18.4% of cancer-related deaths. This disease presents a significant public health challenge, particularly in China, where both the incidence and mortality rates of lung cancer have recently surged.^1^^,^^2^ Many patients with lung cancer do not exhibit symptoms in the early stages and are in the terminal phase at the time of diagnosis, with a five-year survival rate of below 15%.^3^ Although advancements in genetic diagnostics and targeted therapies have improved treatment outcomes, persistent use of chemotherapeutic and targeted drugs frequently leads to drug resistance, undermining these gains.^4^, ^5^, ^6^ Current studies highlight the tumor microenvironment (TME) as a critical factor in the aggressive behavior of tumor cells, and molecular characterization of both the tumor and TME is driving the development of new therapeutic approaches.^7^^,^^8^

**Fig. 1:**
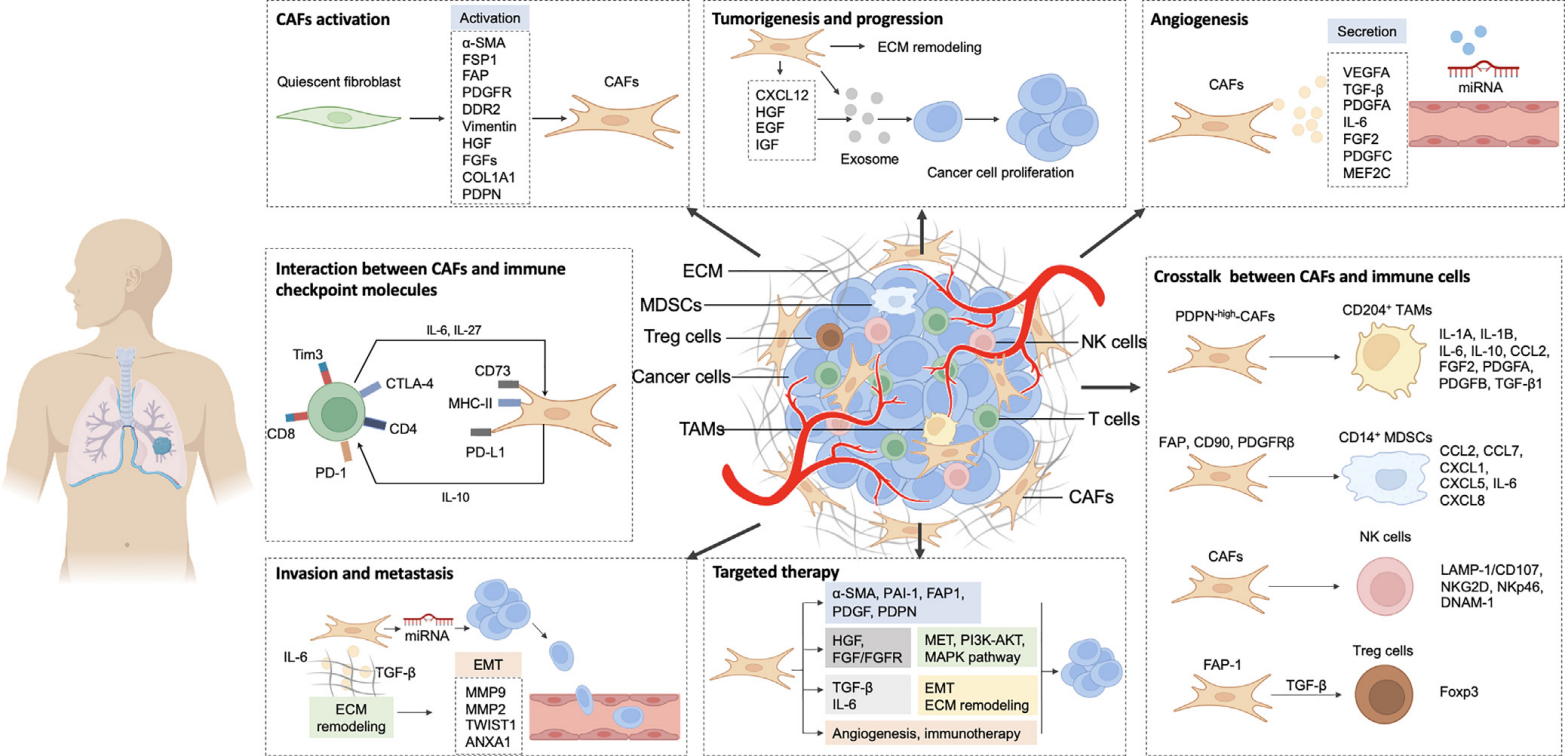
Molecular biomarkers and crucial therapy strategies based on CAFs in lung cancer. ANXA1: Annexin A1; α-SMA: Alpha-smooth muscle actin; CAFs: Cancer-associated fibroblasts; CCL: Chemokine (C-C motif) ligand; COL1A1: Collagen 1 alpha 1; CTLA-4: Cytotoxic T-lymphocyte-associated protein 4; CXCL: Chemokine (C-X-C motif) ligand; DDR2: Discoidin domain receptor tyrosine kinase 2; DNAM-1: DNAX accessory molecule-1; ECM: Extracellular matrix; EGF: Epidermal growth factor; EMT: Epithelial-to-mesenchymal transition; FAP: Fibroblast activation protein; FGFR: Fibroblast growth factor receptor; FGFs: Fibroblast growth factors; FGFR: Fibroblast growth factor receptor; Foxp3: Forkhead box P3; FSP1: Ferroptosis suppressor protein 1; HGF: Hepatocyte growth factor; IGF: Insulin-like growth factor; IL: Interleukin; LAMP-1: Lysosomal associated membrane protein 1; MAPK: Mitogen activated protein kinase; MDSCs: Myeloid-derived suppressor cells; MEF2C: Myocyte enhancer factor 2C; MET: Mesenchymal epithelial transition factor; MHC-II: Major histocompatibility complex II; miRNA: MicroRNA; MMP: Matrix metalloproteinase; NK cells: Natural killer cells; NKG2D: Natural killer cell group 2D; NKp46: Natural cytotoxicity triggering receptor 1; PDGF: Platelet-derived growth factor; PAI-1: Plasminogen activator inhibitor-1; PDGFA: Platelet-derived growth factor A; PDGFB: Platelet-derived growth factor B; PDGFC: Platelet-derived growth factor C; PDGFR: Platelet-derived growth factor receptor; PD-1: Programmed death-1; PD-L1: Programmed death-ligand 1; PDPN: Podoplanin; PI3K-AKT: Phosphatidylinositol 3-kinase/protein kinase B; TAMs: Tumor-associated macrophages; TGF: Transforming growth factor; Tim3: T cell immunoglobulin and mucin domain-containing protein 3; TWIST1: Twist homolog 1; VEGFA: Vascular endothelial growth factor A.

The TME is a complex and heterogeneous cellular milieu comprising tumor cells, vascular vessels, cancer-associated fibroblasts (CAFs), immune cells, the extracellular matrix (ECM), and an array of cytokines secreted by these cells.^9^^,^^10^ The TME plays a pivotal role in various lung cancer processes, including initiation, progression, therapeutic resistance, invasion, metastasis, immune evasion, and responses to targeted therapies; these processes involve a spectrum of altered stromal cells.^11^, ^12^, ^13^ CAFs, a diverse group of cells and primary components of the surrounding stroma,^14^ promote fibrosis and ECM remodeling to enhance deposition and structural changes,^15^^,^^16^ and possess dual roles in tumor dynamics, either by promoting or inhibiting tumor growth through various mechanisms. These cells are crucial for regulating tumor proliferation and metastasis by secreting enzymes and proteins that remodel the ECM and promote angiogenesis, thereby facilitating tumor growth. Studies of CAFs within the TME are essential for identifying therapeutic targets and improving immunotherapy efficacy in lung cancer^17^^,^^18^ (Fig. 1).

Recent studies have increasingly focused on CAFs because of their pivotal roles in lung cancer, as revealed by single-cell RNA sequencing.^19^ CAF subpopulations are important contributors to cancer initiation, progression, and the efficacy of targeted therapies, and thus show potential for enhancing personalized treatment approaches.^20^ However, understanding of the functional heterogeneity of CAFs remains limited, hindering the development of effective treatments. This review presents the latest insights into the biology, function, and heterogeneity of CAFs, emphasizing their vital interactions with lung cancer cells and their implications for future research and clinical applications.

## Origin and biomarkers of CAFs

Fibroblasts, initially identified as key cells in connective tissue repair, can be recruited or activated by tumor cells to become CAFs, resembling the process that occurs in wound healing.^21^ CAFs may originate from multiple sources within the TME: (1) resident fibroblasts, which are prevalent and become activated in response to tumor signals; (2) bone marrow-derived cells, including mesenchymal stem cells or progenitors that migrate to the TME and differentiate into CAFs; (3) epithelial and endothelial cells, which transform into fibroblasts through epithelial-to-mesenchymal transition (EMT) or endothelial-to-mesenchymal transition; and (4) adipocytes, influenced by tumor-derived cellular signals, transdifferentiate into fibroblast-like cells.^22^ Specific biomarkers are critical for distinguishing CAFs from other TME cells (Table 1); these biomarkers include alpha-smooth muscle actin (α-SMA), a marker of myofibroblastic activation;^23^ fibroblast activation protein (FAP), involved in ECM remodeling and tumor support;^24^ platelet-derived growth factor receptor alpha and beta (PDGFRα and PDGFRβ), which facilitate their recruitment and activation;^25^ vimentin, indicating their mesenchymal origin;^26^ fibroblast-specific protein 1, suggesting a transition to a fibroblastic phenotype;^27^ and discoidin domain-containing receptor 2, crucial for ECM organization and promoting collective tumor cell invasion through distinct pathways.^28^

**Table 1:** Representative CAF targeting biomarkers in lung cancer.

CAF marker	Cell types	Expression level in CAFs	Biological functions	References
CAFs biomarkers				
α-SMA	Activated fibroblasts, myofibroblast, and vascular smooth-muscle cells	Upregulated	Activation of myofibroblasts, and maintaining cellular structure and integrity	^32^^,^^33^^,^^73^^,^^93^
FSP1 (S100A4)	Quiescent fibroblasts, epithelial cells, and macrophages	Upregulated	Promotes the activation of fibroblasts and tissue fibrosis	^33^
FAP	Activated fibroblasts, mesodermal cells, immune cells, and cancer cells	Upregulated	ECM remodeling and fibrogenesis	^34^^,^^72^
PDGFRβ	Activated or quiescent fibroblasts, pericytes, vascular smooth muscle cells, skeletal muscles	Upregulated	A receptor tyrosine kinase which upon activation by PDGF stimulates cell proliferation	^30^
Podoplanin	Activated fibroblasts and lymphatic endothelial cell	Upregulated	Induces resistance to EGFR-TKIs in lung cancer with *EGFR* mutation	^36^^,^^73^^,^^74^
Growth factor related to CAFs				
FGF/FGFR	Activated fibroblasts, smooth muscle cells, and macrophages	Upregulated or downregulated	FGFs, angiogenic factors	^42^^,^^43^^,^^54^
IGF2	Activated fibroblasts and osteoblasts	Upregulated	CAFs regulate cancer stemness and cell proliferation through IGF2	^41^
HGF	Activated fibroblasts and mesenchymal cells	Upregulated	HGF regulates cell growth, motility, and morphogenesis by c-Met receptor and is responsible for resistance to EGFR tyrosine kinase inhibitors	^44^^,^^75^^,^^76^
Cytokine related to CAFs				
CXCL12	Activated fibroblasts, BM-MSCs, osteoblasts, and hematopoietic cells	Upregulated	CXCL12 secreted by CAFs promotes metastasis of cancer cells	60, 61, 62
CCL2	Activated fibroblasts, monocytes, and other immune cells	Upregulated	Regulates cellular adhesion and chemotaxis of macrophages, and increases tumor cell extravasation and angiogenesis	^92^^,^^95^^,^^97^
IL-6	Activated fibroblasts and T cells, monocytes, endothelial cells	Upregulated	Promotes anti-inflammatory responses, induces cell proliferation, angiogenesis, EMT, and drug resistance	^31^^,^^35^^,^^81^^,^^82^
TGF-β	Activated fibroblasts	Upregulated	Cytokine that promotes EMT, inhibits antitumor immunity, and promotes resistance to targeted therapies and chemotherapies	^33^^,^^45^^,^^80^^,^^81^^,^^92^
Angiogenesis-related proteins of CAFs				
VEGFA	Activated fibroblasts, endothelial cells, and immune cells	Upregulated	Promotes angiogenesis, increases vascular permeability, promotes invasion and chemoresistance	^50^
PDGFC	Activated fibroblasts, mesenchymal cells	Upregulated	Promotes angiogenesis and inflammation, and increases cell proliferation and migration	55, 56, 57
RNA binding factors				
miR-196a	Activated fibroblasts and cancer cells	Upregulated	MiR-196a promotes the migration and invasion of lung cancer cells	^68^
miR-200	Activated fibroblasts and cancer cells	Downregulated	MiR-200 deficiency promotes lung cancer metastasis by activating Notch signaling in CAFs	^67^
miR-101-3p	Activated fibroblasts and cancer cells	Upregulated	VEGFA is a novel target of miR-101-3p, and CAF-derived VEGFA mediates the effects of miR-101-3p on the migration and invasion of lung cancer cells	^53^
CSCs relative proteins				
CD10	Early B and T lymphoid precursors, smooth muscle cells and myoepithelial cells, and fibroblasts	Upregulated	Marker of common (pre-B) acute lymphocytic leukemias and certain CSCs	^71^
GPR77	Activated fibroblasts and neutrophils	Upregulated	Inflammatory reaction and pro-inflammatory signaling	^71^
CD44	Activated fibroblasts and immune cells	Upregulated	Immunologic activation, cell adhesion, and cell–cell interaction	^92^

α-SMA: Alpha-smooth muscle actin; AKT: Serine/threonine protein kinase B; BM-MSCs: Bone marrow derived mesenchymal stem cells; CAFs: Cancer-associated fibroblasts; CCL2: Chemokine (C-C motif) ligand 2; CSCs: Cancer stem cells; CXCL12: Chemokine (C-X-C motif) ligand 12; ECM: Extracellular matrix; EGFR: Epidermal growth factor receptor; EMT: Epithelial-to-mesenchymal transition; eNOS: Endothelial nitric oxide synthase; FAP: Fibroblast activation protein; FGFs: Fibroblast growth factors; FGFR: Fibroblast growth factor receptor; FSP1: Ferroptosis suppressor protein 1; GPR77: G protein-coupled receptor 77; HGF: Hepatocyte growth factor; IGF2: Insulin-like growth factor 2; IL-6: Interleukin 6; miR: MicroRNA; PDGF: Platelet-derived growth factor; PDGFC: Platelet-derived growth factor C;" and "PDGF: Platelet-derived growth factor; PDGFRβ: Platelet-derived growth factor receptor beta; TGF-β: Transforming growth factor β; TKIs: Tyrosine kinase inhibitors; VEGFA: Vascular endothelial growth factor A.

Analysis of specific biomarkers of CAFs is vital for understanding their roles in tumor progression and developing targeted therapies.^29^^,^^30^ However, these biomarkers are not exclusive to CAFs and are also present in other cell types and healthy tissues. For instance, α-SMA is present in vascular smooth muscle cells, fibroblast-specific protein 1 in macrophages and some cancer cells, FAP in certain tumor-associated macrophages (TAMs), and PDGFRα and PDGFRβ in vascular endothelial cells and multiple tumors.^31^, ^32^, ^33^ The heterogeneity of CAFs, both in their markers and functions, emphasizes the need for further research to identify specific biomarkers and functional subsets.^34^ Moreover, the recruitment of CAFs to tumors, driven by growth factors and cytokines from cancer and immune cells, impacts cancer development, angiogenesis, metastasis, and therapeutic responses. Thus, investigating the heterogeneity and biomarkers of CAFs is essential for understanding the complex dynamics within the TME, and targeting CAFs may provide new insights for cancer treatment.

## Heterogeneity of CAFs in lung cancer

CAFs exhibit diverse phenotypes, origins, and roles and form distinct subgroups within the TME. Advances in single-cell RNA sequencing have improved the understanding of this diversity in various tumor types. Identifying specific CAF subsets and reliable cell-surface markers is crucial for advancing clinical research. Novel biomarkers, including fibroblast growth factors (FGFs), hepatocyte growth factor (HGF), and ECM proteins such as collagen, can be used to distinguish CAF subgroups.^35^ Particularly, FGFs, an important family of polypeptides integral to pulmonary biology, contribute to functional heterogeneity within CAFs, influencing cancer progression and patient responses to treatments such as epidermal growth factor receptor (EGFR) and anaplastic lymphoma kinase tyrosine kinase inhibitors.^36^ Using single-cell RNA-sequencing, Lambrechts *et al*^37^ demonstrated that lung tumors contain five distinct types of fibroblasts, each exhibiting different gene expression and high phenotypic diversity. These results indicated that CAFs have a strong EMT signal that correlates with the expression of a range of ECM proteins (such as collagen 1 alpha 1 [COL1A1], collagen 3 alpha 1 [COL3A1], and collagen 6 alpha 1 [COL6A1]) and transforming growth factor (TGF)-β-associated genes. These insights improved the understanding of the biology of lung cancer CAFs and may lead to improvements in lung cancer diagnosis and therapy. Additionally, Galbo *et al*^38^ conducted an integrated analysis of CAFs from multiple cancer types, identifying six CAF subtypes with specific molecular biomarkers. The authors identified four distinct subpopulations of CAFs in non-small cell lung cancer (NSCLC): NSCLC CAFs 1–4. NSCLC CAFs 1 were classified as desmoplastic CAFs based on the elevated expression of collagen genes (*COL1A1*, collagen 1 alpha 2 (*COL1A2*), *COL3A1*, and collagen 8 alpha 1 (*COL8A1*). NSCLC CAFs 2 closely resembled the myofibroblast-like CAFs described by Lambrechts *et al*,^37^ characterized by elevated expression of the activated fibroblast marker actin alpha 2, smooth muscle (ACTA2), the smooth muscle cell marker myosin heavy chain 11 (MYH11), and markers for platelet derived growth factor (PDGF) signaling. NSCLC CAFs 3 were classified as inflammatory CAFs based on the elevated expression of inflammatory factors, including complement factor D and cytokines interleukin-6 (IL-6), chemokine (C-C motif) ligand 2 (CCL2), and chemokine (C-X-C motif) ligand 2 (CXCL2). NSCLC CAFs 3 were likely non-tumor-derived fibroblasts based on the lack of expression of CAF-related collagens. NSCLC CAFs 4 highly expressed several genes related to translation (*EEF1A1* and *RPL17*) and metabolism and were classified as metabolic CAFs.

Molecular characterization of CAF heterogeneity in lung cancer reveals both differences and similarities compared to those in melanoma and head and neck squamous cell carcinoma. In melanoma, two distinct subtypes of CAFs have been identified: inflammatory-like CAFs, based on the elevated expression of molecules related to complement system (C3 and C1S) and chemokines (chemokine [C-X-C motif] ligand 14, CXCL14), and proliferating CAFs, based on the elevated expression of genes associated with the cell cycle such as marker of proliferation Ki-67 (*MKI67*) and baculoviral IAP repeat containing 5 (*BIRC5*). For head and neck squamous cell carcinoma, the authors identified five distinct sub-populations (head and neck squamous cell carcinoma CAFs 1–5). These cells were classified as: myofibroblast-like CAFs based on the elevated expression of marker genes for activated fibroblasts (ACTA2) and myogenesis (MYH11, myosin light chain 9 [MYL9], myosin light chain kinase [MYLK]); desmoplastic CAFs, based on the elevated expression of markers associated with collagen (COL1A1, COL1A2, and COL3A1) and ECM remodeling (matrix metalloproteinase 11 and 19, MMP11 and MMP19); inflammatory CAFs, based on the elevated expression of complement immunity-related molecules (C3), CAF chemokine marker chemokine (C-X-C motif) ligand 12 (CXCL12), and CXCL14; and normal myofibroblast, displaying elevated expression of myogenic-related genes (myogenic factor 5 and 6, *MYF5* and *MYF6*), with significantly enriched myogenesis Gene Ontology terms; and metabolic CAFs, with elevated expression of ECM-related genes (fibronectin 1, *FN1*; collagen 10 alpha 1, *COL10A1*).

CAF phenotypes are closely linked to outcomes in patients with NSCLC. Comprehensive spatially resolved single-cell imaging mass cytometry analysis was performed on CAFs from a cohort of 1070 patients with NSCLC. The authors identified four patient groups based on the unique spatial distributions of 11 CAF phenotypes, establishing CAFs as independent prognostic factors for patient survival. Notably, the presence of tumor-like CAFs is correlated with poor prognosis. In contrast, inflammatory and interferon-responsive CAFs are associated with an inflamed TME and improved patient survival rates. Furthermore, a high density of matrix CAFs, characterized by low immune infiltration, is inversely related to patient survival.^39^ Multimarker-defined CAF subsets were examined using high-content spatial profiling. The robust associations among CAFs, driver mutations, immune features, and outcomes highlight the critical roles of CAFs in NSCLC progression and underscore the need for further studies to explore their potential as biomarkers or therapeutic targets.^40^ Collectively, these findings emphasize the high molecular diversity of CAFs in lung cancer, a critical factor in tumor progression and immunotherapy resistance. Ongoing research is vital to identify potential CAF markers and classify subtypes for precision-targeted therapies (Table 2).

**Table 2:** Heterogeneity of CAFs and new biomarkers in lung cancer.

Subtypes of CAFs	Biomarkers	Functions	References
Hypoxic CAFs	CAIX	Associations with shorter survival	^39^
SMA CAFs	SMA, FAP, MMP, collagen	Associations with good prognosis	
dCAFs	Ki-67	Associations with relapse	
vCAF	CD146	Predictors of poor prognosis	
iCAFs	CD34, CD248	Associations with longer survival	
Hypoxic tCAFs	CAIX, CD10	Predictors of poor prognosis	
tCAFs	CD10, CD73	Associations with relapse and shorter survival	
ifnCAFs	IDO	Associations with longer survival and good prognosis	
PDPN CAFs	PDPN		
mCAFs	MMP11, SMA, collagen	Predictors of poor prognosis	
Collagen CAFs	Collagen, FN	Associations with relapse and shorter survival	
Subtype I CAFs	HGF^High^ FGF7^High/Low^ p-SMAD2^Low^	Mediated EGFRi resistance through activating MET, highest EGFRi rescue capacity	^20^
Subtype II CAFs	HGF^Low^ FGF7^High^ p-SMAD2^Low^	Intermediate EGFRi rescue capacity	
Subtype III CAFs	HGF^Low^ FGF7^Low^ p-SMAD2^High^	Chemoattractant properties for T lymphocytes and monocytes	
Cluster 1 of CAFs	COL10A1	EMT regulation	^37^
Cluster 2 of CAFs	COL4A1, ACTA2, MEF2C, MYH11, or ITGA7	Myogenesis and angiogenesis	
Cluster 4 of CAFs	PLA2G2A	EMT regulation	
Cluster 5 of CAFs	MMP3, RGS5	Metabolic regulation	
Cluster 6 of CAFs	FIGF, elastin	–	
Cluster 7 of CAFs	CCL2, RGS5	Metabolic regulation	
Myofibroblast-like CAFs	ACTA2, MYH11, MCAM, TAGLN, and MYLK	Associations with smooth muscle contraction and vascular wound healing	^38^
Desmoplastic CAFs	COL1A1, COL3A1, MMPs, TGF-β	ECM remodeling	
Inflammatory-like CAFs	CFD, C3, CXCL14, and CXCL12	Associated with the effectiveness of immune therapy	
Metabolic CAFs	EEF1A1 and RPL17	Associated with metabolism	

ACTA2: Alpha-smooth muscle actin 2; C3: Complement C3; CAFs: Cancer-associated fibroblasts; CAIX: Carbonic anhydrase IX; CD146: Cluster of differentiation 146; CFD: Complement factor D; COL1A1: Collagen 1 alpha 1; COL3A1: Collagen 3 alpha 1; COL4A1: Collagen 4 alpha 1; COL10A1: Collagen 10 alpha 1; CXCL12: Chemokine (C-X-C motif) ligand 12; CXCL14: Chemokine (C-X-C motif) ligand 14; dCAFs: Dividing CAFs; ECM: Extracellular matrix; EEF1A1: Eukaryotic translation elongation factor 1A1; EGFRi: Epidermal growth factor receptor inhibitors; EMT: Epithelial-to-mesenchymal transition; FAP: Fibroblast activation protein; FGF7: Fibroblast growth factor 7; FN: Fibronectin; HGF: Hepatocyte growth factor; iCAFs: Inflammatory CAFs; IDO: Indoleamine 2,3-dioxygenase; ifnCAFs: Interferon-response CAFs; ITGA7: Integrin subunit alpha 7; mCAFs: Matrix CAFs; MCAM: Melanoma cell adhesion molecule; MEF2C: Myocyte enhancer factor 2C; MMP: Matrix metalloproteinase; MYH11: Myosin heavy chain 11; MYLK: Myosin light chain kinase; p-SMAD2: Phospho-mothers against decapentaplegic homolog 2; PDPN: Podoplanin; PLA2G2A: Phospholipase A2, group IIA; RPL17: Ribosomal protein L17; RGS5: Regulator of G protein signaling 5; ‌SMA: Smooth muscle actin; TAGLN: Transgelin; tCAFs: Tumour-like CAFs; TGF-β: Transforming growth factor-β; vCAF: Vascular CAFs; -: Not available.

## Role of CAFs in lung cancer

### Effects of CAFs on tumorigenesis and progression

CAFs play a pivotal role in lung cancer development through mechanisms such as paracrine signaling, exosome transfer, physical interaction, and ECM modification.^41^^,^^42^ Horie *et al*^43^ demonstrated that lung cancer cells proliferate more rapidly when co-cultured with patient-matched CAFs, underscoring the role of CAFs in promoting lung cancer cell growth *in vitro*. Chen *et al*^44^ established a sustainable primary culture that highlighted the function of CAFs in tumor cell proliferation, particularly through the secretion of chemokines and growth factors such as CXCL12 and HGF. Furthermore, co-culturing CAFs with cancer cells is effective in tumor promotion, notably through the secretion of fibroblast growth factor 2 (FGF2).^45^^,^^46^ Lung fibroblasts interact with NSCLC cells via the HGF/MET pathway, which greatly enhances NSCLC tumorigenesis and progression.^47^ Additionally, CAF-derived exosomes play a crucial role in NSCLC progression.^48^^,^^49^ Moreover, the ECM, profoundly influenced by CAFs, is essential for the initiation and progression of lung cancer.^50^ As key components of the TME, CAFs secrete ECM remodeling enzymes and proteins that drive tumor proliferation and progression.^51^

### Effects of CAFs on angiogenesis

Angiogenesis, which is critical for cancer development, is influenced by both tumor and stromal cells within the TME. Research indicates that CAFs promote tumor angiogenesis by secreting proangiogenic factors and ECM remodeling proteins.^52^^,^^53^ Specifically, CAFs produce key molecules, such as vascular endothelial growth factor A (VEGFA),^54^ CXCL12,^55^ FGF2,^56^^,^^57^ and platelet-derived growth factor C (PDGFC),^58^, ^59^, ^60^ to enhance angiogenesis and tumor growth (Table 1). Beyond their interactions with cancer cells, CAFs engage with other stromal cells via cytokine release and exosomal transmission.^61^ Interestingly, tumor angiogenesis can also proceed in a vascular endothelial growth factor (VEGF)-independent manner, with CAF overexpression in lung cancer facilitating the proliferation of vascular endothelial cells.^62^ Moreover, mesenchymal stem cells from lung tumors, expressing CAF markers such as VEGF, CXCL12, and transforming growth factor β1 (TGF-β1), may differentiate into CAFs and contribute to neoplastic progression and metastasis.^63^ Qian *et al*^64^ identified a unique set of angiogenesis-related genes in CAFs, including EGF like domain multiple 6 (*EGFL6*), angiopoietin2 (*ANGPT2*), and platelet-derived growth factor A (*PDGFA*). Notably, CAFs are the primary sources of CXCL12 and CXCL14 in the TME, which strongly influence angiogenesis and tumorigenesis. Myocyte enhancer factor 2C (MEF2C), which is highly enriched in CAFs, plays a crucial role in regulating neoangiogenesis. These insights underscore the potential of targeting CAFs as a therapeutic strategy against lung cancer through modulation of angiogenic factors.

### Effects of CAFs on invasion and metastasis

A growing body of research indicates that cancer invasion and metastasis are influenced by the TME, particularly through interactions with CAFs.^65^ CAFs contribute to cancer cell migration, invasion, and metastasis by synthesizing growth factors and cytokines and remodeling the ECM. The interaction between CAFs and the ECM is dynamic, with the ECM acting as a reservoir for secreted proteins and facilitating tumor invasion and metastasis.^66^ Recent studies reported that CAFs enhance lung cancer cell migration and invasion by inducing EMT, as evidenced by changes in E-cadherin and vimentin expression. CAFs activate the janus kinase 2/signal transducer and activator of transcription 3 (JAK2/STAT3) pathway via IL-6 secretion, promoting metastasis.^67^ In invasive lung adenocarcinoma, the proportion of CAFs and matrix metalloproteinase 9 (MMP9) expression is increased, suggesting a significant role in tumor invasion.^68^ Matrix metalloproteinase 2 (MMP2) status in CAFs is also an important prognostic factor in NSCLC.^69^ Emerging research on microRNAs revealed that CAFs influence NSCLC cell migration and invasion via miR-101-3p mediated VEGFA secretion and the serine/threonine protein kinase B/endothelial nitric oxide synthase (AKT/eNOS) pathway. Additionally, miR-200 deficiency in CAFs and miR-196a activation are linked to lung cancer metastasis.^70^^,^^71^ Moreover, CD10+ G protein-coupled receptor 77 (GPR77)+ CAFs support tumor formation and maintain cancer stem cell (CSC) characteristics, reinforcing the role of CAFs in tumor aggression and resistance.^72^ Therefore, targeting CAFs is crucial in anti-metastasis therapy for lung cancer.

### Effects of CAFs on therapeutic resistance and targeting strategies for CAFs in lung cancer

The primary goals of identifying critical biomarkers and understanding the functional heterogeneity of CAFs in the TME are to develop strategies that target essential cancer vulnerabilities. Further studies are required to identify specific CAF markers suitable for targeted therapy. Given the important role of CAFs in the tumorigenesis, progression, angiogenesis, and metastasis of lung cancer, formulating therapeutic strategies to target CAFs is vital. The high heterogeneity of CAFs in lung cancer necessitates extensive examination of various biomarkers to decipher this diversity.^73^ Understanding both the biomarkers and subtypes of CAFs is crucial for enhancing cancer treatment and addressing therapeutic resistance (Fig. 1).

The expression of α‐SMA, a stromal marker, in CAFs is linked to poor survival and chemotherapeutic resistance in NSCLC, as it upregulates inflammatory factors and chemokines. Inhibition of plasminogen activator inhibitor‐1, which is secreted by CAFs and other cells, reduces α‐SMA expression, increasing CAF apoptosis and affecting chemotherapy sensitivity.^74^ FAP1, another CAF marker, may serve as a predictive marker of the efficacy of treatments targeting the tumor stroma or modulating the immune environment. Additionally, FAP1 expression in CAFs has been explored as a prognostic indicator in NSCLC, with higher FAP1 levels generally associated with poorer patient outcomes.^75^ Podoplanin (PDPN), which is overexpressed in CAFs, contributes to ECM remodeling and poor clinical outcomes, suggesting its role as a biomarker for CAF-targeted therapy.^76^ Yoshida *et al*^77^ suggested that PDPN-positive CAFs are crucial in primary resistance to *EGFR*-tyrosine kinase inhibitors, presenting them as potential targets for combination therapy. NSCLC CAFs co-cultured with *EGFR* mutant cancer cells activated the MET signaling pathway and/or FGF receptor, rescuing *EGFR*-positive cancers. Thus, CAFs may protect cancer cells by activating other tyrosine kinase receptor-mediated signaling, thereby bypassing the need for EGFR signaling. This study also highlighted the role of FGF/FGF receptor, in addition to HGF, in NSCLC.^23^ CAFs inhibit the response of MET-amplified NSCLC cells to MET kinase inhibitors in an HGF-dependent manner, with HGF activating the phosphorylation of MET and EGFR via signaling pathways.^78^

CAFs play a crucial role in mediating drug resistance through various mechanisms, such as ECM remodeling and cytokine-mediated signaling^79^ by forming a physical barrier that hinders drug efficacy.^80^^,^^81^ TGF-β, overexpressed in CAFs along with other ECM proteins and cytokines including FGFs and bone morphogenetic proteins, plays a dual role in promoting resistance.^82^^,^^83^ The interaction of IL-6 with TGF-β, particularly on lung cancer cells, underscores its important role in tumor progression,^84^ exemplified by IL-6-mediated chemoresistance through TGF-β-induced EMT.^85^ Moreover, TGF-β1 receptor inhibition treatment in lung CAFs reduces their ability to confer resistance to EGFR inhibitors. HGF secretion by CAFs is also a critical factor in EGFR-tyrosine kinase inhibitor resistance.^86^ Additionally, targeting the PDGF/PDGFR signaling pathway in the interaction between CAFs and cancer cells undergoing EMT is a promising therapeutic approach.^87^^,^^88^

CAF-mediated angiogenesis in the TME may facilitate tumor cell growth and therapy resistance.^82^ For advanced lung cancer, treatment strategies increasingly incorporate molecular targeted therapies like angiogenesis inhibition, with the VEGF/VEGFR pathway central to this approach. Bevacizumab, which targets VEGF, was approved by the Food and Drug Administration in 2006 for treating advanced NSCLC,^89^ and several VEGFR tyrosine kinase inhibitors have been approved in combination with chemotherapy for metastatic NSCLC.^90^^,^^91^ CAFs are a primary source of VEGF/VEGFA in tumor-associated angiogenesis. miR-101-3p, which is downregulated in lung CAFs, can suppress CAF activation and target VEGFA, influencing lung cancer cell migration and invasion.^50^ Moreover, other factors like PDGFs and FGFs are linked to resistance against EGFR and VEGF-targeting agents.^92^, ^93^, ^94^

CSCs, which are known for their high tumorigenicity and chemoresistance, are significantly affected by CAFs. CD10+ GPR77+ CAFs contribute to tumor formation and chemoresistance in CSCs by activating the nuclear factor-κB pathway.^72^ Moreover, CD44 abundantly expressed on CAFs is implicated in sustaining CSCs stemness, which is crucial for lung cancer cell drug resistance.^95^ Thus, targeting CAFs may be a promising strategy to combat anticancer therapeutic resistance, offering new avenues for developing innovative anticancer treatments.

## Correlation between CAFs and tumor immunology in lung cancer

The lung TME contains a variety of cell types contributing to the formation of an immunosuppressive microenvironment, which may play pivotal roles in lung cancer initiation, progression, metastasis, and responses to immunotherapy.^96^ CAFs, identified based on the expression of fibroblast-associated markers such as FAP, CD90, and PDGFRβ, play a central role in influencing the tumor immune response. They achieve this effect by synthesizing and secreting ECM proteins, ECM-remodeling enzymes, cytokines, and chemokines that modulate the recruitment and activation of tumor immune cells including TAMs, myeloid-derived suppressor cells (MDSCs), and various lymphoid cell populations.^97^

### Immune suppression by CAFs

High PDPN expression in CAFs has been linked to CD204+ TAM infiltration of NSCLC cells. The PDPN-high group revealed significant upregulation of IL-1A, IL-1B, IL-6, IL-10, CCL2, FGF2, PDGFA, PDGFB, and TGF-β1, which contributed to establishing an immunosuppressive microenvironment in patients with lung cancer. Additionally, studies of tumor-infiltrating CD204+ TAMs in NSCLC following induction therapy revealed a correlation between CD204+ TAMs and the survival and prognosis of patients with NSCLC.^98^^,^^99^

Lung cancer CAFs exert their immunosuppressive effects by secreting chemokines, including CCL2, chemokine (C-C motif) ligand 7 (CCL7), chemokine (C-X-C motif) ligand 1, 5, and 8 (CXCL1, CXCL5, CXCL8), and IL-6, which promote the differentiation of CD14+ monocytes into immunosuppressive MDSCs. These CAF-induced MDSCs further impede the proliferation of CD8+ T-cells by upregulating the expression of nicotinamide adenine dinucleotide phosphate oxidase type 2 (NOX2) and indoleamine 2,3-dioxygenase 1 (IDO1), resulting in greatly elevated production of reactive oxygen species.^100^ Furthermore, CAFs impact the functions of natural killer cells by inhibiting their proliferation, impairing their cytotoxic capacity, and inducing degranulation. In NSCLC, CAFs exert immunosuppressive effects on natural killer cells by reducing the surface levels of lysosomal-associated membrane protein-1/CD107, natural killer cell group 2D (NKG2D), natural cytotoxicity triggering receptor 1 (NKp46), and DNAX accessory molecule-1 (DNAM-1).^101^

Cytotoxic T cells, an essential component of antitumor immune responses, are more commonly found in the stromal regions of lung cancer.^102^ Activated T cells enhance the expression of major histocompatibility complex II, the co-inhibitory molecules ‌programmed death-ligand 1 (PD-L1) and CD73 on the surface of CAFs, leading to increased production of IL-6 and IL-27. CAFs, in turn, regulate T cell functions by upregulating the expression of multiple co-inhibitory receptors, including CD39, programmed death-1 (PD-1), and T cell immunoglobulin and mucin domain-containing protein 3 (Tim3), and by inducing the production of IL-10 by T cells.^103^ Consequently, the impairment of T cell function mediated by CAFs can strongly affect anti-tumor immunity.^104^ It is currently accepted that CAFs participate in T cell exclusion from tumor nests in human lung cancer.^105^ Four main CAF populations were identified using single-cell RNA sequencing coupled with multiplex imaging in a large cohort of lung tumors. Two of these populations, MYH11 + α-SMA+ CAFs in early stage tumors and FAP + α-SMA+ CAFs in advanced tumors, were associated with CD3+ and CD8+ T cell exclusion from the tumor nests. These populations collectively coordinate a distinct structural tissue arrangement characterized by dense and aligned fiber deposition in contrast to T cell-permissive CAFs.

This investigation revealed that diverse CAF populations exhibited greater heterogeneity than did traditionally recognized CAFs. These findings present novel therapeutic targets for enhancing the response to cancer immunotherapy. Current research has entered the clinical trial stage (NCT06024538 and NCT06107608) based on tumor-associated fibroblasts and the application of immunotherapy in lung cancer. Related clinical trials are presented in Table 3. The future of clinical medicine for treating lung cancer depends on the outcomes of these large-scale trials.

**Table 3:** Clinical trials of CAFs in lung cancer.

Target	Name	Mechanism	Phase	Number of participants	Primary endpoint	Study completion date	References
FAP	FAPI	Prevents FAP expression	A pilot study	60	Diagnosis	2025-12-31	NCT05543954
PDGFR	Lucitanib	Prevents the expressions of FGFR 1–3, VEGFR 1–3, and PDGFR α/β	Phase II study	18	Treatment	2016-09	NCT02109016
FGF/FGFR	Erdafitinib, JNJ-42756493	Reduces signaling downstream of FGFR	Phase II trial	22	Treatment	2022-10-14	NCT03827850
IGF1R	GSK1838705A, linsitinib	Inhibits the expression of IGF1R	Preclinical study	–	–	–	^113^
VEGF/VEGFR	Pazopanib	Prevents signaling from CAFs; multi-target receptor tyrosine kinase inhibitor of VEGFR-1, -2, -3, PDGFR-alpha and -beta and c-kit	Phase I and phase II trials	35	Treatment	2008-04	NCT00367679
TGF-β	Galunisertib, nivolumab	Prevents CAFs expression	Phase I and phase II trials	41	Treatment	2020-07-08	NCT02423343

CAFs: Cancer-associated fibroblasts; EGFR: Epidermal growth factor receptor; FAP: Fibroblast activation protein; FGF: Fibroblast growth factor; FGFR: Fibroblast growth factor receptor; FAPI: Fibroblast activation protein inhibitor; IGF1R: Insulin-like growth factor 1 receptor; PDGFR: Platelet-derived growth factor receptor; TGF-β: Transforming growth factor beta; VEGF: Vascular endothelial growth factor; VEGFR: Vascular endothelial growth factor receptor; c-kit: c-kitproto oncogeneprotein; -: Not available.

CAFs can directly or indirectly upregulate PD-L1 in tumor cells and immune cells within the TME. CAFs secrete factors such as CXCL2, which increase PD-L1 expression in lung cancer cells, thus promoting an immunosuppressive environment. CAFs were found to indirectly influence tumor immunity by increasing PD-L1 expression in lung adenocarcinoma cells.^106^ CAFs also alter the function of T cells by producing cytokines such as TGF-β and IL-10, which inhibit T cell activation and proliferation. This effect dampens the adaptive immunity and interferes with the effectiveness of cytotoxic T-lymphocyte-associated protein 4 (CTLA-4) inhibitors, which aim to boost T-cell activity by blocking the CTLA-4 checkpoint.^107^ The presence of CAFs in the TME has been linked to resistance to immune checkpoint inhibitors. The fibrotic matrix produced by CAFs can physically impede T cell infiltration into the tumor, limiting the access of immune cells reactivated by therapies targeting PD-1/PD-L1 and CTLA-4. Additionally, secretion of VEGF by CAFs can reduce treatment effectiveness by promoting angiogenesis and further enhancing the barrier to immune cell infiltration.^108^ Understanding the interaction between CAFs and immune checkpoints has led to the exploration of combination therapies that target both fibrotic and immunosuppressive components of the TME.

### Immune promotion by CAFs

Traditionally, desmoplastic reactions are considered a barrier to immune cell infiltration. However, emerging evidence indicates that CAFs promote immunity by secreting chemokines and cytokines that attract and activate immune cells. For instance, specific CAF subsets in lung cancer were found to significantly increase the production of chemokine (C-X-C motif) ligand 13 (CXCL13) by both CD4+ and CD8+ T cells, which chemoattract cytotoxic T cells. This result suggests that certain CAFs facilitate an immune response against the tumor by promoting the infiltration and activity of T cells within the TME.^109^ Additionally, PD-L1 expression on CAFs is reversibly regulated by environmental stimuli, including IFN-γ from activated lymphocytes. In non-metastatic NSCLC, PD-L1 expression on CAFs suggests the induction of anti-tumor immune responses, contributing to better prognosis after surgery and impacting the interaction between tumor cells and the immune system.^110^ Moreover, recent studies identified a subset of CAFs that exhibit a so-called “inflammatory” phenotype, characterized by the production of pro-inflammatory cytokines such as IL-6. These cytokines contribute to the recruitment and activation of immune cells, potentially enhancing antitumor immunity.^111^ However, the same factors can also promote tumor growth and angiogenesis, highlighting the dual role of CAFs in lung cancer. The heterogeneity of CAFs in lung cancer demonstrates the complexity of their interactions with the immune system. Although some CAF subsets promote immune responses, others can suppress or evade immune recognition, contributing to immune tolerance and tumor progression. Understanding these nuances is crucial for developing targeted therapies that harness the potential of the immune system to combat lung cancer.

In summary, CAFs play a pivotal role in shaping the immune response in patients with lung cancer, and targeting CAFs shows promise for enhancing the efficacy of immunotherapy in modulating CAF activity. Identification of specific CAF subtypes and their distinct roles in immune regulation has provided a foundation for targeted interventions. Strategies include targeting CAF-derived signaling molecules, disrupting the immunosuppressive CAF phenotype, and promoting antitumor immune responses.^112^ Novel therapeutic agents and biologics aimed at reprogramming CAFs to create an immune-favorable milieu are currently under investigation.

## Challenges of targeting CAFs in NSCLC treatment

### Heterogeneity of CAFs

The high functional and phenotypic heterogeneity of CAFs within the TME complicates the development of treatments that uniformly target CAFs without affecting non-target cells. Different subtypes of CAFs may have distinct roles in tumor progression and response to therapy; therefore, targeting one population may inadvertently promote the activity of another, potentially exacerbating the disease.

### Risk of tumor promotion

Whereas some CAFs support tumor growth and metastasis, others may suppress tumor progression. The dual role of CAFs poses a significant risk; inhibiting CAFs that are naturally tumor suppressive may unintentionally facilitate cancer progression, leading to worse patient outcomes.

### Compromised normal tissue function

CAFs share characteristics with fibroblasts involved in normal wound healing and tissue repair processes. Thus, targeting CAFs may disrupt normal healing and fibrosis processes in healthy tissues, leading to unintended side effects, such as impaired wound healing or enhanced fibrotic responses.

### Impact on immune response

CAFs play complex roles in modulating the immune environment within the TME by suppressing or facilitating these responses. Targeting CAFs may impair the delicate immune balance of the TME, potentially leading to reduced immunosurveillance of tumor cells or increased autoimmunity.

### Development of resistance

As with many targeted therapies, there is a concern that tumors may develop resistance to treatments targeting CAFs. The tumor may adapt by upregulating alternative pathways that compensate for the inhibited CAF function or by recruiting and modifying new CAFs from precursor cells.

With the continued development of single-cell omics, heterogeneous CAF populations and novel biomarkers related to targeted therapy for lung cancer must be precisely defined. Therefore, studies are needed to understand the function and heterogeneity of CAFs and to identify specific biomarkers to develop novel targeted therapies against lung cancer.

## Conclusions and future perspectives

Cancer cell-induced reprogramming of the lung TME contributes to lung carcinogenesis. In this review, we summarized recent progress regarding the heterogeneous subpopulations and diverse functions of CAFs in lung cancer. We also explored the mechanisms underlying CAF-mediated regulation of tumorigenesis, progression, angiogenesis, invasion and metastasis, resistance to therapy, tumor immune suppression, and targeted therapy response in lung cancer (Fig. 1). Understanding the role of CAFs in lung cancer will improve the understanding of their impact on the disease. Future preclinical and clinical studies should focus on systematically defining, comprehensively characterizing, and functionally validating CAF subpopulations to facilitate the development of novel diagnostic and therapeutic approaches for lung cancer.

Although progress has been made in understanding the role of CAFs in lung cancer, knowledge of these cells lags behind that of tumor cells in lung cancer. Although most well-established CAF biomarkers can be used to identify CAFs, none of these markers is specific for CAFs in the lung TME. For several decades, CAFs have been recognized as partners of cancer cells that can function as positive or negative regulators of tumor growth. Whether this property can be attributed to different subtypes of CAFs or the same CAF subpopulations working in a context-dependent manner and at different stages of tumor progression remains unknown.

## Declaration of competing interest

No potential conflict of interest was reported by the authors.
